# Beyond the Bedside in the Age of Artificial Intelligence (AI): Preserving the Humanistic Core of Nursing

**DOI:** 10.17533/udea.iee.v44n1e01

**Published:** 2026-03-15

**Authors:** Eddieson Pasayan

**Affiliations:** 1 Ph.D. Department of Nursing Administration and Education, College of Nursing, King Khalid University, Abha, Saudi Arabia. Email: edpasayan@gmail.com. Corresponding author. https://orcid.org/0000-0003-1257-3175 Department of Nursing Administration and Education, College of Nursing King Khalid University Abha Saudi Arabia edpasayan@gmail.com

## Introduction

The incorporation of AI into healthcare has grown exponentially over a very short period of time and is changing how healthcare services are provided. Using AI in nursing can have an enormous impact by improving both the quality of and efficiency of patient care.^1^ While AI could be considered a threat to the nursing field because of its rapid growth in delivering health care, there is as well an opportunity to improve patient care by using AI in nursing. AI can positively affect the nursing profession through enhancing the efficiency and quality of care provided to patients.^2^ The biggest issue that will affect the nursing profession's adaptation to new technology will be defining what nursing is and where AI will fit into nursing's evolving role with AI and other emerging technologies. As such, it allows the nursing profession to continue to lead the way in all areas of the nursing profession. While the growing use of artificial intelligence is often viewed as a threat, it actually presents a significant opportunity for the field to innovate and enhance the delivery of high-quality patient care.^3^^,^^4^

### Humanistic Aspects of Nursing

There are tremendous opportunities to utilize AI to enhance the quality and efficiency of patient care in the clinical environment, and to reduce the amount of routine and administrative work associated with nursing.^5^^)^ Therefore, AI has the ability to provide nurses with more time to develop a therapeutic relationship with their patients. Predictive algorithms utilized in AI systems can assist nurses in providing high-quality patient care. This is by analyzing vast amounts of critical patient data and enabling nurses to make informed and timely interventions based on the information provided by the algorithm.^1^^,^^5^ For example, virtual nursing assistants, which may be integrated into smart infusion pumps or used as a standalone device, may enable nurses to intervene and save a patient's life up to hours before they are recognized as being in a crisis by the clinician. In addition to utilizing predictive analytics, chatbots, and virtual companions, AI systems have the capability to support patient monitoring, scheduling, and record keeping.^6^ The integration of AI algorithms into the various areas of nursing practice - including patient assessment, nursing care plan development, patient education, and nursing administrative responsibilities - has the potential to further foster the relationship between patients and nurses who typically do not have the time to develop these relationships.^6^ While implementing AI in a functional way, nursing is able to preserve many of the fundamental elements of caring (i.e. physical presence and humanity), more so than any other profession. Nursing-specific AI tools focus on areas of practice that are unique to nursing (e.g. patient education, care coordination, and holistic assessments).^7^ The rapid growth of AI in healthcare presents a large opportunity to improve the care that patients receive. In terms of nursing, the primary benefit of using AI will be to decrease the number of repetitive tasks which are a major part of a nurse’s role. This occurs through the use of AI algorithms within many aspects of nursing practice allowing for a relationship to form between the patient and nurse - typically there is no time to make this happen.

### AI in Nursing Practice

There are multiple challenges for the ethical and professional development of nursing practice as it quickly adopts AI. This includes such as the issue of professional responsibility, professional autonomy, and others. Algorithmic bias is a significant concern with the adoption of AI into nursing practice, because of concerns about the transparency of the algorithms, distrust of the algorithms, security of patient data, and fairness. ^3^ There are also ethical considerations about AI in terms of how machine learning algorithms will be developed to make AI systems ethically sound, and to develop systems that have the same capacity to think intelligently as humans do. For example, the training data that is used to train AI must be representative of all patient populations, if the data does not include equal representation of all patient populations then the AI could potentially disproportionately affect the most vulnerable and marginalized populations.^8^^,^^9^ In addition to protecting patients from the negative consequences of AI, the practice of nursing must be defined to ensure that the professional decisions of nurses are not replaced by the decisions of the AI system. Therefore, a fundamental responsibility of nurses is to define the ethical boundaries of using AI, and the criteria for the use of AI in patient care.^10^ Additionally, the nurse must determine whether AI-generated prompts result in harm, and determine the ethical responsibilities of the nurse when the AI prompts result in harm. As a result of these determinations, critical analysis will be required to identify the criteria for determining what type of interventions can be provided by a nurse, or by an AI device. Therefore, critical thinking will be essential to distinguish between the role of a nurse and the role of an AI device in providing patient-centered, appropriate health care services. As a result of the rapid adoption of AI into nursing practice, the nurse must continue to remain at the top of the hierarchy of technical skills and use of the technology, and the nurse's professional responsibility must never be usurped by the technology.^10^

### Ethically integrating AI in practice

The integration of AI in nursing will require an unprecedented level of interdisciplinary collaboration among nurses, Artificial Intelligence/Information Technology (AI/IT) specialists, ethicists, and policy-makers to appropriately integrate AI algorithms into nursing practice.^11^ Nurses can no longer simply be passive consumers of AI technology and must become equal partners in the development of AI tools that are useful in clinical settings.^12^ Also, nurses need to address the complex nature of nursing processes while resolving the healthcare needs of diverse populations and nursing systems. Advocacy and ethical support from the public and professional nursing community will provide the only sustained means to protect patient safety while utilizing nursing practices supported by AI technologies. Lastly, understanding the nature of nursing and its evolving applications of AI will significantly influence the adaptation of nursing to these emerging technologies and remain at the forefront of the profession.

### Bridging the gap

Nursing education has not kept pace with the rapidly evolving artificial intelligence (AI) technologies. At present, there is a disconnect between the curriculum of a nursing program and the real world of a nurse in practice. This requires an immediate response because of the need for drastic changes in nursing programs' curricula to "bridge the gap". In fact, dramatic changes to nursing curricula have the potential to affect all nursing fields equally. While many nursing curricula are lacking in AI-related areas of general health informatics (e.g., ethics, algorithmic bias, data literacy, and critical evaluation of AI output), education has a responsibility to prepare the new clinician in the integration of AI and machine learning into their nursing practice.^3^^,^^13^ This through learning about data literacy and how to critically evaluate AI outputs. This educational preparation will allow the new clinician to efficiently utilize and question the AI-generated recommendations. Further, there is an imperative for structured training for the current nursing workforce in the new curriculum.^14^ Without this education/training, nurse-initiated AI projects will be developed without expertise and ultimately, will result in negative impacts on patient outcomes.
